# Acrylamide-targeting renal miR−21a−5p/Fibrotic and miR122-5p/ inflammatory signaling pathways and the role of a green approach for nano-zinc detected via *in silico* and *in vivo* approaches

**DOI:** 10.3389/fphar.2024.1413844

**Published:** 2024-07-17

**Authors:** Leena S. Alqahtani, Manal E. Alosaimi, Amany Abdel-Rahman Mohamed, Yasmina M. Abd-Elhakim, Tarek Khamis, Ahmed E. Noreldin, Ali H. El-Far, Badriyah S. Alotaibi, Mohammed Ageeli Hakami, Naief Dahran, Nouf A. Babteen

**Affiliations:** ^1^ Department of Biochemistry, College of Science, University of Jeddah, Jeddah, Saudi Arabia; ^2^ Department of Basic Sciences, College of Medicine, Princess Nourah Bint Abdulrahman University, Riyadh, Saudi Arabia; ^3^ Department of Forensic Medicine and Toxicology, Faculty of Veterinary Medicine, Zagazig University, Zagazig, Egypt; ^4^ Department of Pharmacology, Faculty of Veterinary Medicine, Zagazig University, Zagazig, Egypt; ^5^ Laboratory of Biotechnology, Faculty of Veterinary Medicine, Zagazig University, Zagazig, Egypt; ^6^ Department of Histology and Cytology, Faculty of Veterinary Medicine, Damanhour University, Damanhour, Egypt; ^7^ Key Laboratory of Epigenetics and Oncology, The Research Center for Preclinical Medicine, Southwest Medical University, Luzhou, China; ^8^ Department of Biochemistry, Faculty of Veterinary Medicine, Damanhour University, Damanhour, Egypt; ^9^ Department of Pharmaceutical Sciences, College of Pharmacy, Princess Nourah Bint Abdulrahman University, Riyadh, Saudi Arabia; ^10^ Department of Clinical Laboratory Sciences, College of Applied Medical Sciences, Al-Quwayiyah, Shaqra University, Riyadh, Saudi Arabia; ^11^ Department of Anatomy, Faculty of Medicine, University of Jeddah, Jeddah, Saudi Arabia

**Keywords:** Moringa, zinc oxide nanoparticles, kidney, TNFRS-1A, TIMP-3

## Abstract

**Introduction:** Any disruption in renal function can have cascading effects on overall health. Understanding how a heat-born toxicant like acrylamide (ACR) affects kidney tissue is vital for realizing its broader implications for systemic health.

**Methods:** This study investigated the ACR-induced renal damage mechanisms, particularly focusing on the regulating role of miR-21a-5p/fibrotic and miR-122-5p/inflammatory signaling pathways via targeting Timp-3 and TP53 proteins in an *In silico* preliminary study. Besides, renal function assessment, oxidative status, protein profile, and the expression of renal biomarkers (Timp-1, Keap-1, Kim-1, P53, TNF-α, Bax, and Caspase3) were assessed in a 60-day experiment. The examination was additionally extended to explore the potential protective effects of green-synthesized zinc oxide nanoparticles (ZNO-MONPs). A four-group experiment including control, ZNO-MONPs (10 mg/kg b.wt.), ACR (20 mg/kg b.wt.), and ZNO-MONPs + ACR was established encompassing biochemical, histological, and molecular levels. The study further investigated the protein-binding ability of ZNO and MONPs to inactivate caspase-3, Keap-1, Kim-1, and TNFRS-1A.

**Results:** ZNO-MONPs significantly reduced ACR-induced renal tissue damage as evidenced by increased serum creatinine, uric acid, albumin, and oxidative stress markers. ACR-induced oxidative stress, apoptosis, and inflammationare revealed by biochemical tests, gene expression, and the presence of apoptotic nuclei microscopically. Also, molecular docking revealed binding affinity between ACR-BCL-2 and glutathione-synthetase, elucidating the potential mechanisms through which ACR induces renal damage. Notably, ZNO-MONPs revealed a protective potential against ACR-induced damage. Zn levels in the renal tissues of ACR-exposed rats were significantly restored in those treated with ACR + ZNO-MONPs. In conclusion, this study establishes the efficacy of ZNO-MONPs in mitigating ACR-induced disturbances in renal tissue functions, oxidative stress, inflammation, and apoptosis. The findings shed light on the potential renoprotective activity of green-synthesized nanomaterials, offering insights into novel therapeutic approaches for countering ACR-induced renal damage.

## 1 Introduction

One of the heat-processing toxicants is acrylamide (ACR), which stands as a prominent environmental contaminant, imparting a range of systemic toxic effects on individuals exposed either through occupational activities or dietary sources (Semla et al., 2017; Dahran et al., 2023). Serving as the foundational monomer for polyacrylamide synthesis, ACR poses a risk of work-related disorders in various industrial settings, such as plastics, paper, cosmetics manufacturing, wastewater treatment, and drinking water purification (Sun et al., 2020). Particularly concerning is the impact on public health due to dietary ACR intake, a byproduct formed during the high-temperature heating processes of tobacco and carbohydrate-rich foods (Baskar and Aiswarya, 2018). Notably, ACR, characterized by high water solubility, can easily diffuse and traverse different organs in the body, including the liver and kidneys (Wang et al., 2017).

Accumulating findings from animal studies highlighted the detrimental impact of ACR exposure on various tissues, including the liver, nervous system, reproductive organs, and kidneys (Rajeh and Al-Dhaheri, 2017; Mahfouz et al., 2023; Mostafa-Hedeab et al., 2023). The study conducted by Nasralla et al. (2021) revealed that ACR-induced oxidative stress in hepatic and renal tissues was accompanied by activated apoptosis. Another investigation focusing on the renal effects of ACR exposure unveiled significant microarchitectural alterations, such as glomerular renal abnormalities characterized by shrinkage, distortion of glomeruli, wrinkling of basement membranes, and widening of urinary spaces (Dasari et al., 2018; Moawad et al., 2019). Additionally, necrotic tubular cells displayed cytoplasmic vacuolation, accompanied by the presence of desquamated epithelial cells within the tubular lumen (Moawad et al., 2019).

The transformation of metals to a nanoscale dimension brings about significant alterations in their chemical, physical, and optical properties. Metallic nanoparticles (NPs), distinguished by their exceptional surface, optical, chemical, biological, resonance, catalytic, and electronic characteristics, have become a focal point of existing research (Kelly et al., 2003). This emphasis extends notably to biosynthesis and novel applications (Dikshit et al., 2021; Khater et al., 2023). Notably, zinc oxide nanoparticles (ZnONPs), approved by the Food and Drug Administration, exhibit a spectrum of beneficial properties, including anticancer, anti-inflammatory, anti-diabetic, antibacterial, antifungal, larvicidal, and wound healing attributes (Kirthi et al., 2011; Malaikozhundan et al., 2020; Chelladurai et al., 2021; Hassan et al., 2021). Concurrently, the utilization of plants for green synthesis with metallic nanoparticles has gained substantial traction (Shreyash et al., 2021; Mohamed et al., 2023). This preference stems from the superior reduction and stabilization capabilities exhibited by plant phytochemicals (Mostafa-Hedeab et al., 2023). Plant extracts, particularly from sources like M. Oleifera, have successfully served as effective reducing agents, converting inorganic Zn into ZnONPs. *Moringa Olifera* (*M. oleifera;*MO) leaf extract, rich in flavonoids such as myricetin, quercetin, kaempferol, isorhamnetin, or rutin, along with phenolic acids, has demonstrated significant success in this regard (Mohamed et al., 2019). The Moringa tree’s fresh leaves further contribute to its appeal as a source of carotenoids like lutein, β-carotene, and zeaxanthin, complemented by a high content of vitamins C and A (Mahmood et al., 2010).

The primary objective of this study is to elucidate the potential nephroprotective effects of green-synthesized ZnONPs utilizing MO leaf extract (ZNO-MONPs) against ACR-induced nephrotoxicity in a rat model. The investigation aimed to comprehensively understand the underlying mechanisms at both structural and functional levels within renal tissue. This has been achieved through the assessment of oxidative stress markers, renal function, and renal tissue level of Zn.Nevertheless, at the genetic level, we planned to evaluate the expression patterns of genes, including Kim-1, Keap-1, Timp-1, and other inflammatory genes. Furthermore, we explored via an *in silico* study to predict the most common interacting microRNA that will inhibit the preselected genes (Timp-3 and P53), and then the expression of miR-21a-5p and miR-122-5p was assessed. Additionally, the histopathological examination was conducted to quantify the extent of renal damage induced by ACR exposure and to assess the potential ameliorative role of ZNO-MONPs. The molecular docking approach employed to unravel the intricate protein interactions involving renal tissue and ACR, as well as the interactions between various proteins related to renal damage and ZNO-MONPs were also assessed. This integrative approach aimed to provide a comprehensive understanding of the toxicity mechanisms and therapeutic potential, facilitating the development of a new effective strategy to mitigate ACR-induced nephrotoxicity.

## 2 Materials and methods

### 2.1 Aqueous extract and *Moringa oleifera* green synthesis with ZnO NPs

The leaves of MO were acquired from the Moringa Scientific Egyptian Society of, National Research Center Egypt Okechukwu et al. (2013). After washing with distilled water, and air-dried, using a milling machine at a high speed they are ground into a powder. Approximately 10 g of the MO powder was mixed with 90 mL of pure water and boiled in a water bath at 60°C for 1 hour with gentle stirring. The greenish extract was separated and cooled. A 20 mL aqueous solution of 1% zinc acetate was added to the previously prepared extract (10 mL in 80 mL DW). The mixture was heated and constantly mixed for about 4 hours, with pH justification during the process. The green synthesis process followed the procedure outlined by Naseer et al. (2020). The resulting ZNO-MONPs were characterized using techniques like transmission electron microscopy, FTIR, dynamic light scattering, and energy dispersive X-ray analysis (EDXA), as detailed in the earlier study by Dahran et al. (2023).

### 2.2 Experimental design

*Sprague Dawley* rats (40 male rats) (12 weeks of age, 154.25 ± 0.30 g) were acquired from the Animal Housing Unit for Lab animals, Faculty of Veterinary Medicine, Zagazig University. Prior to commencing the study, a 2-week adaptation period was granted to the animals. All procedures and care for the animals adhered to the guidelines outlined by the National Institutes of Health in the United States. The research protocol gained an approval ID (Approval No. ZU-IACUC/2/F/101/2022) from the Ethics of the Animal Use Research Committee at Zagazig University in Egypt. The animal experiments applied in the present research comply with the Animal Research: Reporting of *in Vivo* Experiments (ARRIVE) guidelines (Percie du Sert et al., 2020). Rats were housed in a laboratory environment with free access to standard granulated rodent food and water under controlled conditions (25°C, 12 h light: 2 h night). The rats were segregated into four groups, each involving 10 rats/treatment. In this experimental study, four distinct groups of rats were established to investigate the impact of different treatments over a 60-day period. The Control group functioned as the baseline, with each rat being administered 1 mL of distilled water via a gastric tube. The ZNO-MONPs group involved gavaging rats with ZNO-MONPs (10 mg/kg b.wt) following the method outlined by (Akintunde J. K. et al., 2021). The ACR-group (Sigma-Aldrich Co., St. Louis, MO, United States) via gastric gavage at a dose of 20 mg/kg b.wt, as per the protocol detailed by Mahfouz et al. (2023). The ACR + ZNO-MONPs group, where rats received both ACR and ZNO-MONPs with 60-min intervals between administrations. The ACR dose selected in the current study is based on our later studies, which acknowledged the toxicity signs in liver tissue of exposed rats by Mahfouz et al. (2023). Also, several studies correlated the meta-analysis data that established dose-response relationships, aiding in the identification of a dose that is both relevant to human exposure and induces measurable nephrotoxic effects. The selected dose of 20 mg/kg b.wt. is likely to fall within the range observed in human exposures during a few years of his life as it was found that the Food and Agriculture Organization/World Health Organization (FAO/WHO) stated that nutritional ACR exposure can fluctuate between 0.3 and 0.8 μg/kg/BW/day (World Health Organization, 2002), contributing to the translational validity of the experimental design.

### 2.3 Sampling

Intraperitoneal (IP) pentobarbital sodium (100 mg/kg b.wt) was applied for induction of anesthesia of rats at the terminal point of the exposure period. Blood samples were obtained from the orbital vessels of all experimental groups to isolate serum samples, which were then stored at −20°C for kidney function analysis. Subsequently, euthanasia was carried out, and each rat’s kidney was extracted, rinsed in physiological saline, and segmented. One segment was homogenized (at 4°C for 15 min at 664 g) for antioxidant and MDA estimation. The next segment was preserved in formalin (10% neutral buffered) for subsequent microscopical and IHC. The last two segments were frozen for zinc concentration assessment in renal tissue and qRT-PCR. The determination of the sample size employed the Resource equation method, as carefully outlined in the study by Charan and Kantharia (2013) available at https://www.ncbi.nlm.nih.gov/pmc/articles/PMC3826013/. The application of this equation, along with consideration of attrition percentage, indicated that a group size of 10 rats is appropriate, and further additions would not contribute to statistical significance. Consequently, the number of rats per group was adjusted to 10 rats.

### 2.4 Analysis of kidney Zn concentration

Varying concentrations were utilized for preparing standard and calibration curves were derived from the essential chemicals needed. All glassware and plastic materials underwent a thorough distilled water cleaning, immersion in 10% HNO_3_, and finally, deionized water to rinse the equipment. 1 g of tissue (Kidney) was wet-digested following the method outlined by Cho et al. (2010).

### 2.5 Assessment of kidney function and protein profile in the serum and protein

The quantification of urea and creatinine concentrations in serum was performed using the techniques outlined by Coulombe and Favreau (1963) and Larsen (1972), respectively. Additionally, the uric acid content in serum samples was assessed utilizing the methodology established by Barham and Trinder (1972). In accordance with the protocol introduced by Badawi (1971), electrophoresis was employed to separate and analyze total serum proteins, globulins, and albumin.

### 2.6 Biochemical examination of oxidative stress markers in the kidney tissue

The kidney tissue homogenate underwent analysis for malondialdehyde (MDA) using a Bio diagnostic kit (Dokki, Giza, Egypt), CAT. No. MD 25 29, following the method outlined by Ohkawa et al. (1979). In this process, an acidic solution at 95°C facilitated the interaction between thiobarbituric acid and MDA for 30 min, resulting in a thiobarbituric acid reactive product with a pink hue. The absorbance of this product was quantified at 534 nm. Additionally, the total antioxidant capacity (TAC) was determined using a kit (CAT. NO. TA 25 13, Bio-diagnostic Co. Dokki, Giza, Egypt), developed according to Koracevic et al. (2001) protocol. The antioxidants in the sample reacted with a known concentration of exogenously added hydrogen peroxide (H2O2). The ability of the antioxidants to neutralize a specific concentration of H2O2 was measured, and the remaining H2O2 concentration was determined calorimetrically through an enzyme reaction converting 3,5-dichloro-2-hydroxy benzensulphonate into a coloured product. Cleaved-Caspase-3 levels in kidney tissue were assessed using rat ELISA kits procured from MyBioSource (San Diego, CA, United States; Catalog #MBS018987).

The investigation, in accordance with the methodologies detailed by Wang and Joseph (1999) and Siddiqui et al. (2010), evaluated the production of reactive oxygen species (ROS) in the kidney samples. This process involved the application of 2, 7-dichlorofluorescin diacetate (DCFH-DA), which reacts with ROS to generate the fluorescent compound dichlorofluorescein (DCF). After homogenizing the tissue samples, DCFH-DA was introduced, and the samples were shielded from light prior to examination under a fluorescence microscope. The microscope parameters included excitation and emission wavelengths of 485 nm and 528 nm, respictively, at ×20 magnification. ROS quantification utilized ImageJ software, with the calculation of corrected total cell fluorescence (CTCF) derived by subtracting the product of the selected cell’s area and the mean fluorescence of the background from the integrated density.

### 2.7 Expression pattern of genes by qRT-PCR

The QIAamp RNeasy Mini kit which is produced by Qiagen, Germany, GmbH is used for extraction of RNA from kidney samples. Adhered to the manufacturer’s directions for the QIAamp RNeasy Mini kit (Qiagen, Germany, GmbH) and following Abdelbaky et al. (2022), total RNA isolation is applied. Target genes, including Kim-1, Timp1, TNF-α, P53, Caspase-3, NRF-2, BAX, and Keap-1, were amplified using primers obtained from Metabion (Germany), as detailed in Table 1. Additionally, the primer sequences for micro-RNA 21a-5P and micro-RNA 122-55P, along with their respective targets, are presented in Table 1.

**TABLE 1:** Primers sequences, accession number, and product size for the quantitative RT-PCR for the analyzed genes in the renal tissue.

Target gene	Forward primer		Reverse primer	bp	Accession no.
GAPDH	GCA​TCT​TCT​TGT​GCA​GTG​CC		TAC​GGC​CAA​ATC​CGT​TCA​CA	74	NM_017008.4
Kim-1	AGC​ACA​TTC​TCC​AGG​AAG​CC		GCC​ACA​CAT​GGT​GAC​AGA​GA	165	NM_173149.2
Timp-1	ACG​CTA​GAG​CAG​ATA​CCA​CG		AGA​AAG​CTG​TCT​GTG​GGT​GG	126	NM_053819.1
Casp-3	GAG​ACA​GAC​AGT​GGA​ACT​GAC​GAT​G		GGC​GCA​AAG​TGA​CTG​GAT​GA	147	NM_012922.2
TNF-α	GGC​TTT​CGG​AAC​TCA​CTG​GA		GGG​AAC​AGT​CTG​GGA​AGC​TC	164	NM_012675.3
Keap-1	CTG​TGA​CAC​TTC​TCC​TGG​GG		GAG​AAG​CAG​GAA​CCA​GGC​AT	159	NM_057152.2
P53	CCC​CTG​AAG​ACT​GGA​TAA​CTG​T		TCT​CCT​GAC​TCA​GAG​GGA​GC	75	NM_030989.3
Bax	GAA​CCA​TCA​TGG​GCT​GGA​CA		GGG​TCC​CGA​AGT​AGG​AAA​GG	102	NM_017059.2
NRF-2	GGT​TGC​CCA​CAT​TCC​CAA​AC		CAG​GGC​AAG​CGA​CTG​AAA​TG	124	NM_001399173.1
mir-122-5p	AAC​CGG​TGG​AGT​GTG​ACA​AT		GTC​GTA​TCC​AGT​GCA​GGG​T		
mir-21a-5p	AAG​CGA​CCT​AGC​TTA​TCA​GAC​T		GTC​GTA​TCC​AGT​GCA​GGG​T		
miRNA Stem-loop					
mir-122-5p RT		GTC​GTA​TCC​AGT​GCA​GGG​TCC​GAG​GTA​TTC​GCA​CTG​GAT​ACG​ACC​AAA​CA			
mir-21a-5p RT		GTC​GTA​TCC​AGT​GCA​GGG​TCC​GAG​GTA​TTC​GCA​CTG​GAT​ACG​ACT​CAA​CA			

GAPDH: Glyceraldehyde 3-phosphate dehydrogenase; kidney injury molecule −1(Kim-1), TIMP, Metallopeptidase Inhibitor 1(Timp-1), Casp-3, tumor necrosis factor (TNF-α), Kelch Like ECH, Associated Protein 1(Keap-1), Tumor protein p53 (P53), Nuclear factor erythroid 2-related factor 2 (NRF-2).

For the real-time PCR reaction, primers were used in a 25-µL setup. The reaction was conducted in a StepOne Real-Time PCR machine, and the program determined amplification curves and ct values. Herein, a set of six appropriate housekeeping genes, including (GAPDH, HRPRT-1, UBC, SDHA, ARBP, and actin-b) are selected using the freely availablesoftware GeNorm (https://genorm.cmgg.be), for normalization and quantification purposes. The stability of each housekeeping gene was checked via a statistical test (ANOVA) for the CT values of all tested genes, and raw data was included in the (Supplementary Figure S1, S2). The analysis revealed a non-significant difference in the expression patterns among the experimental groups with GAPDH, as indicated by a minimal standard deviation (SD). These findings suggest that the housekeeping gene GAPDH exhibits stable expression and remains unaffected by the treatment type. The "Ct” method and calculation were applied as described by Livak and Schmittgen (2001).

### 2.8 Molecular docking assessment

#### 2.8.1 Instruments and tools

In this study, we used the Windows 10 Pro operating system and Intel (R) Core (TM) DDR4 RAM i7-5500U CPU @ 2.40 GHz.

#### 2.8.2 Ligand preparation

https://pubchem.ncbi.nlm.nih.gov/PubChem database which is used to obtain the three-dimensional structures of acrylamide and zinc oxide as SDF format. Also, *M.oleifera’s* bioactive compounds were retrieved from LOTUS (https://lotus.naturalproducts.net/) database.

#### 2.8.3 Protein preparation

The three-dimensional structures of rats’ caspase-3, Keap1, KIM-1, tumor necrosis factor receptor superfamily member 1A (Tnfrsf1a), B-cell lymphoma 2 (Bcl2), and glutathione synthetase were retrieved from (https://www.uniprot.org/) known as the UniProt database. Target proteins were prepared for docking using UCSF Chimera software by removing water and ligand molecules present in the protein structures, along with target protein energy minimization.

#### 2.8.4 Molecular docking analysis and visualization

Target proteins were docked with ligands using InstaDock (Mohammad et al., 2022). Finally, the protein-ligand interactions were visualized by the BIOVIA discovery visualization 2024 Client software.

### 2.9 Microscopical architectural assessment

Following the euthanasia of rats, kidney samples were collected and preserved in neutral buffered formaldehyde for a duration of 48 h. Subsequently, the preserved samples underwent processing through the conventional paraffin embedding technique. Sections measuring 4 μm in thickness were then stained using hematoxylin and eosin (H and E) as earlier explained by Bancroft and Layton (2013). In brief, an experienced non-biased pathologist blindly examined all slides from all groups under 40 high-power fields randomly choosing 10 fields, from each group, and obtained the micrograph using a digital camera (Leica EC3, Leica, Germany). The detected lesions represented by lymphocytic infiltration, renal tubule necrosis, and renal glomeruli necrosis were estimated and scored by a non-biased pathologist according to the criteria reported by Gibson-Corley et al. (2013) [Score scale: 0 = normal; 1 = mild lesions; 2 = moderate lesions; 3 = severe; 4 = extremely severe]. The attained data for the scores were averaged and denoted with graphs. Also, some sections were stained with Masson’s trichrome for total collagen detection. The Fiji image analyzer, an extension of ImageJ software developed by the National Institutes of Health in Bethesda, MD, United States, was employed to accurately calculate the percentage of area occupied by collagen. Ten images were randomly chosen from each group and processed to separate collagen (green) and acid fuchsin (magenta) colors. A standardized color threshold was then applied across all images to determine collagen distribution percentages uniformly (Vis et al., 2000).

### 2.10 Statistical analysis

The samples underwent assessment for normal distribution using the Shapiro-Wilk test, and the homogeneity of variance, indicating equal variances, was verified through the Levene test on the dataset prior to analysis. To conduct a one-way analysis of variance (ANOVA) and assess the statistical significance of intergroup differences, GraphPad Software version 8 (San Diego, CA, United States) was employed (Flyverbom, 2019). Following this, Tukey’s multiple range *post hoc* test was applied for group comparisons in cases where normality assumptions were satisfied. For the histopathological scores, a Kruskal–Wallis test followed by Dunn’s multiple comparisons test was applied. The standard error (SE) for each group was reported, and statistical significance was established at a *p-value < 0.05*.

## 3 Results

### 3.1 Effects on kidney functions and protein profile

According to the data presented in Table 2, the group exposed to ACR exhibited a substantial (*p < 0.001*) rise in serum levels of creatinine, urea, uric acid, and albumin by 588.72%, 255.36%, 260.28%, and 54.78%, respectively, when compared to the control group. Conversely, ACR exposure led to a significant (*p < 0.001*) decrease in the levels of total proteins and globulin in the serum of exposed rats by 7.15% and 40.82%, respectively, compared to the control. However, oral administration of MO.ZNO-NPs significantly (*p < 0.001*) attenuated the ACR-induced increases in creatinine, urea, uric acid, and albumin by 71.5%, 57.2%, 49.02%, and 14%, respectively, relative to the control values, with significant differences compared to the ACR-exposed group. Furthermore, the combination therapy of ZNO-MO NPs with ACR resulted in a significant (*p < 0.001*) increase in globulin levels by 55.06%, as depicted in Table 2. However, no significant effect was observed in the total protein levels between the ACR and the combination therapy groups.

**TABLE 2:** Effect of zinc oxide nanoparticles synthesized from Moringa oleifera extract (ZNO-MONPs) oral dosing on the kidney function and serum proteins of adult male *Sprague Dawley* rats exposed to acrylamide (ACR) for 60 days.

Estimated parameters	Control	ZNO-MONPs	ACR	ACR + ZNO-MONPs
Creatinine (mg/dL)	0.39 ± 0.02	0.42 ± 0.03	2.49 ± 0.09*	0.71 ± 0.02*#
Urea (mg/dL)	45.70 ± 0.65	48.10 ± 1.16	162.4 ± 2.4*	69.50 ± 2.86*#
Uric acid (mg/dL)	1.41 ± 0.06	1.24 ± 0.03	5.08 ± 0.21*	2.59 ± 0.05*#
Total protein (mg/dL)	6.43 ± 0.18	6.89 ± 0.06*	7.76 ± 0.11	6.12 ± 0.05#
Albumin (mg/dL)	3.56 ± 0.05	3.53 ± 0.06	5.51 ± 0.05*	3.80 ± 0.10#
Globulin (mg/dL)	2.87 ± 0.09	2.90 ± 0.04	2.25 ± 0.03*	2.32 ± 0.07*#

Means within the same row carrying different superscripts are significantly different at *p* < 0.05. Values shown are means ± SE. n = 10 group. *p < 0.05 vs. control, and # *p < 0.05* vs. ACR.

### 3.2 The detected Zn concentration in the kidney tissue

**FIGURE 1:**
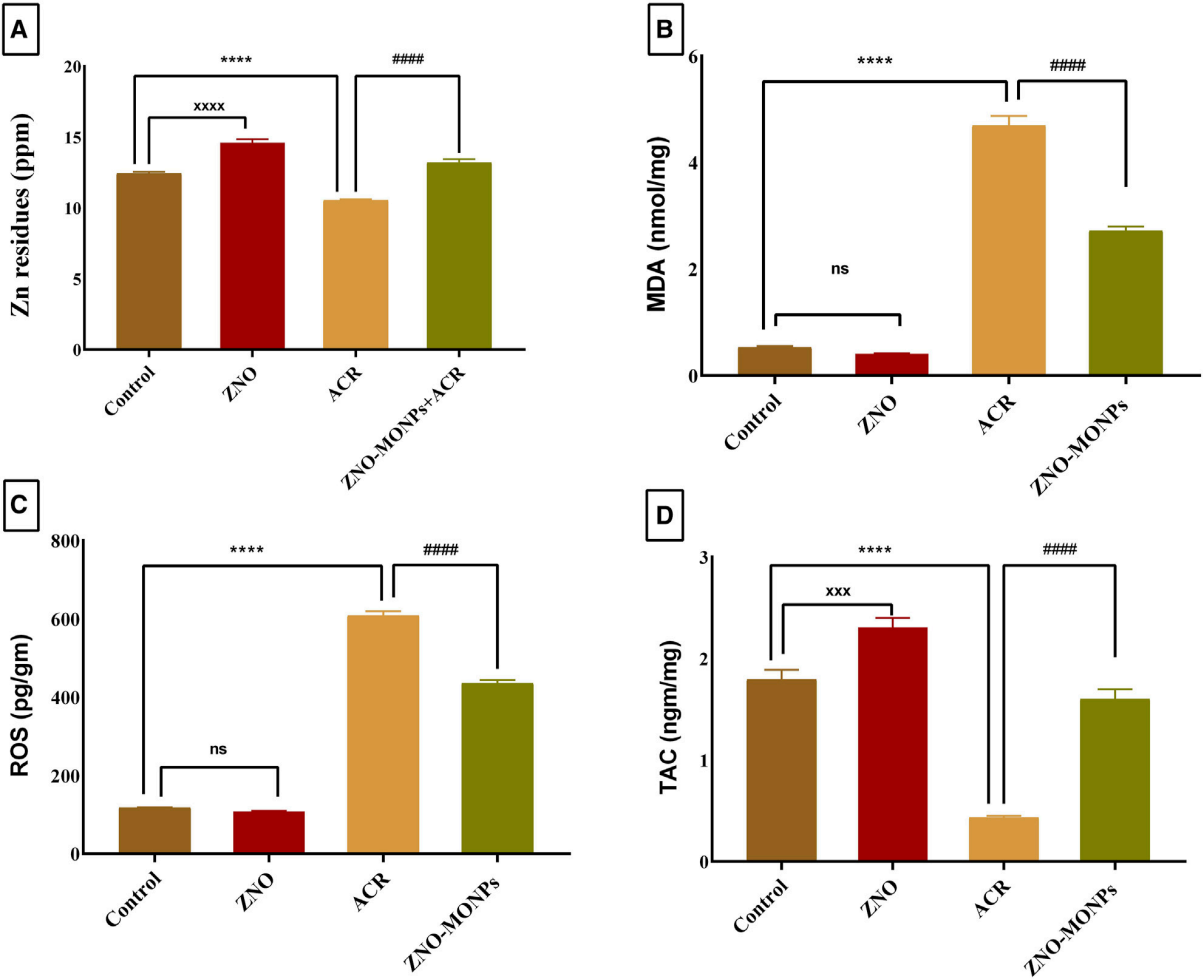
Effect of *Moringa olifera*-mediated zinc oxide nanoparticles (ZNO-MONPs) and/or acrylamide (ACR) exposure on Zn residues **(A)** and Malonaldehyde (MDA) **(B)**, Reactive oxygen species (ROS) **(C)**, and Total antioxidant capacity (TAC) **(D)** in the kidney tissues of all experimental groups including control of adult male *Sprague Dawley* rats for 60 days. Bars represent the mean ± SE. n = 10. ****p < 0.001 vs. control, #### *p < 0.001* vs. ACR and ZNO-MONPs xxxx *p < 0.001* vs. control.

Compared to the control group, rats administered ZNO-MO-NPs for 60 days exhibited a 17.44% increase in zinc deposition in kidney tissue (Figure 1A). In contrast, the concentration of Zn in the kidneys of the ACR group significantly decreased by 15.19% compared to the control (*p < 0.001*). Co-administration of ZNO-MONPs with ACR substantially restored Zn content in rat kidneys (*p < 0.001*), indicating a 9.65% increase compared to the ACR group. Notably, there was a significant accumulation of ZNO-MONPs by 27.79% compared to the ACR-exposed group after 60 days.

### 3.3 Effects on oxidative stress markers in renal tissue

The renal tissue levels of MDA and ROS (Figures 1B,C) in the ACR group exhibited a significant (*p* < 0.001) increase, reaching 784.37% and 416.37%, respectively, compared to those in the control group, indicating severe renal oxidative stress. Additionally, ACR treatment resulted in a substantial decrease in the renal level of TAC by 75.71% compared to the control group (Figure 1D). Notably, our study revealed a trend towards the normalization of oxidative stress parameters. Upon supplementation with ZNO-MONPs in rats, there was a notable reduction in the content of ROS and MDA in renal tissue by 42.12% and 28.45%, respectively. In comparison, TAC significantly increased by 267.77% compared to the values observed in the ACR group.

### 3.4 Estimation of the expression pattern of apoptotic, inflammatory, and kidney injury-related genes via qRT-PCR

**FIGURE 2:**
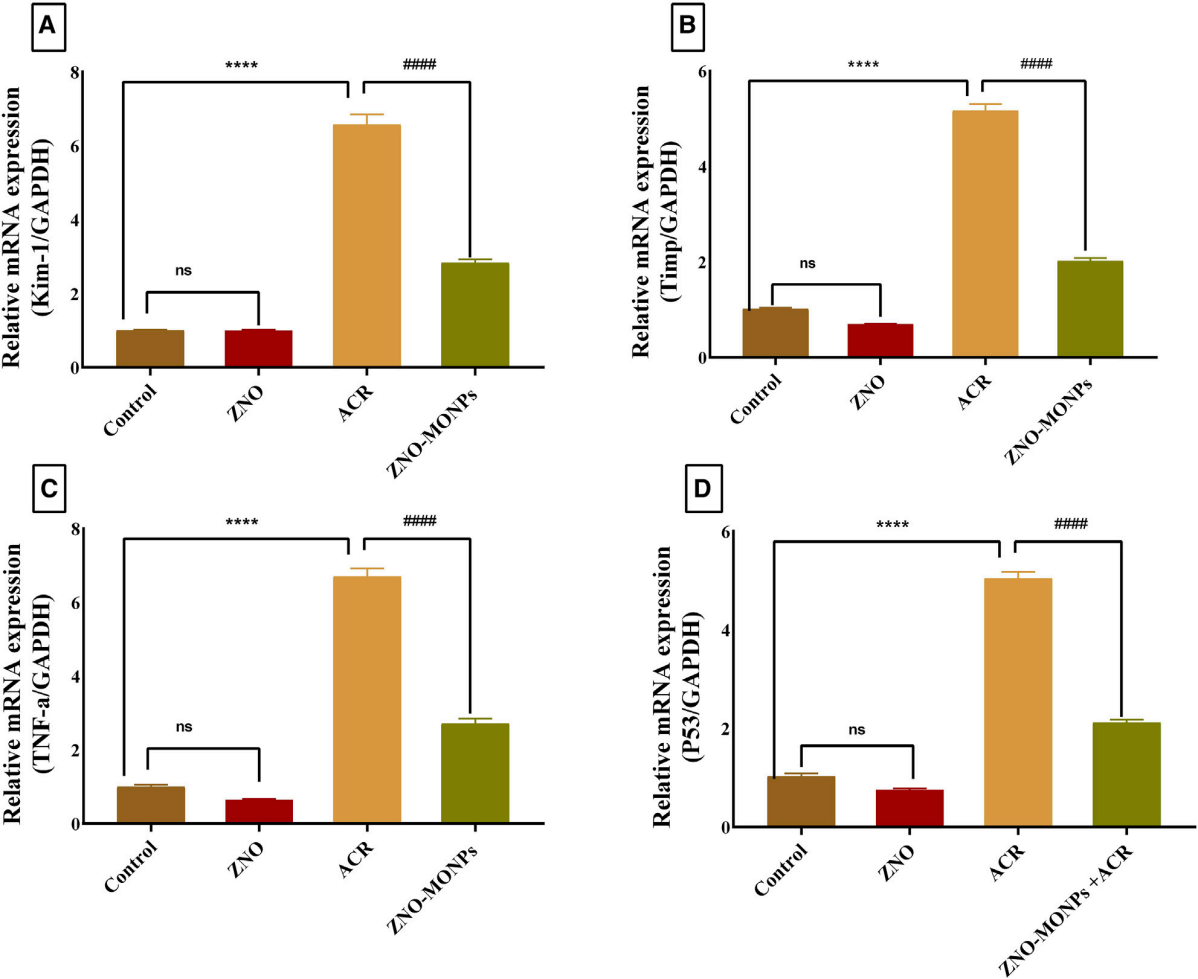
Effect of *Moringa olifera*-mediated zinc oxide nanoparticles (ZNO-MONPs) and/or acrylamide (ACR) exposure on the expression of kidney injury molecule −1(Kim-1)) **(A)**, Metallopeptidase Inhibitor 1(Timp-1) **(B)**, tumor necrosis factor (TNF-α) **(C)**, tumor protein p53 (P53) **(D)** of all experimental groups including control of adult male *Sprague Dawley* rats for 60 days. Bars represent the mean ± SE. n = 10. ****p < 0.001 vs. control, #### *p* < 0.001 vs. ACR.

**FIGURE 3:**
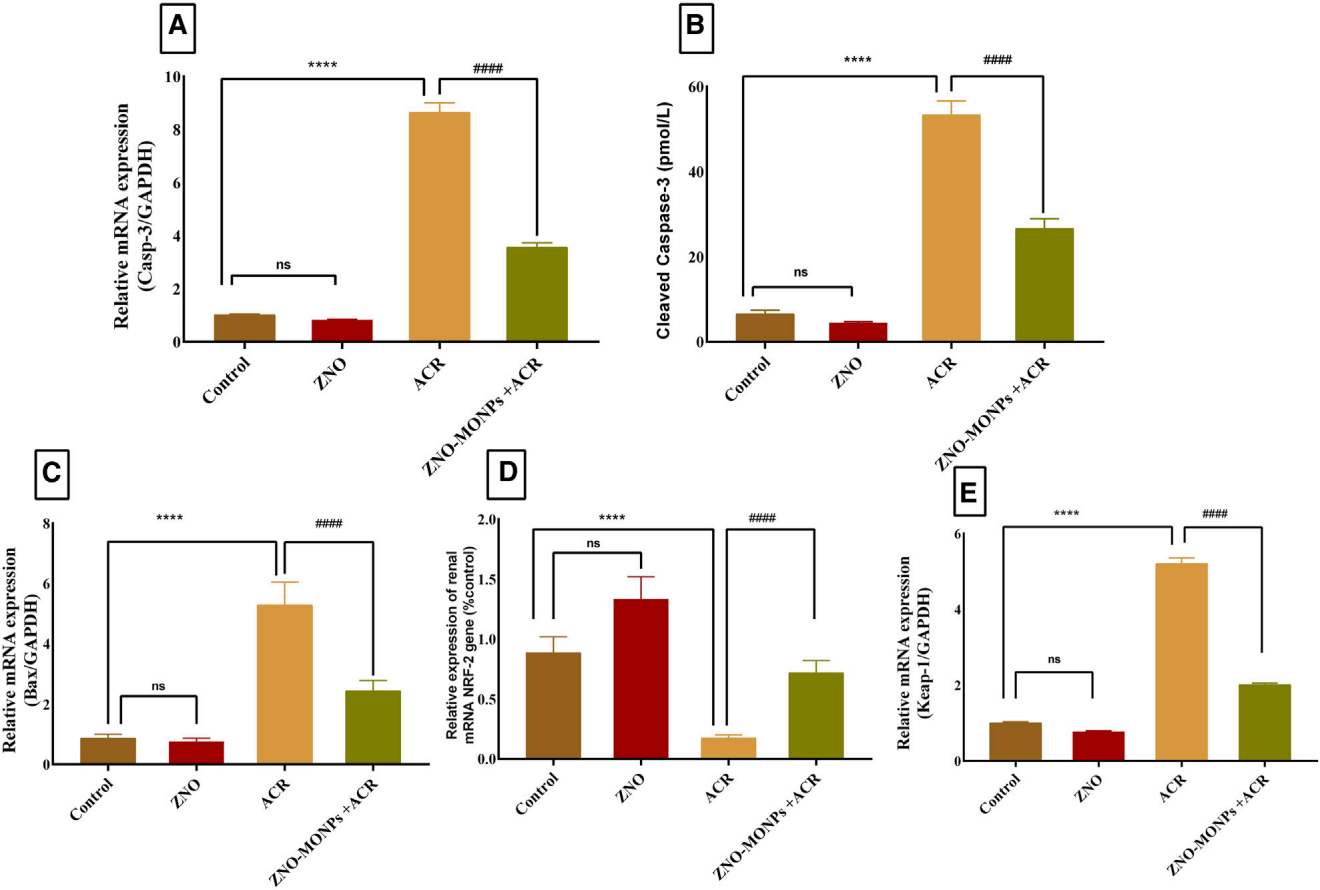
Effect of Moringa olifera-mediated zinc oxide nanoparticles (ZNO-MONPs) and/or acrylamide (ACR) exposure on the expression of kidney Caspase-3 **(A)**, Cleaved Caspase-3 **(B)**, Bcl-2-associated X protein (BAX) **(C)**, Nuclear factor erythroid 2-related factor 2 (NRF-2) **(D)**, Kelch Like ECH Associated Protein 1 (Keap-1) **(E)** of all experimental groups including control of adult male Sprague Dawley rats for 60 days. Bars represent the mean ± SE. n = 10. **** *P* < 0.001 vs control, #### *P* < 0.001 vs ACR.

The presented data in Figure 2 for gene expression analysis of different genes in the renal tissue revealed significant (*p < 0.001*) upregulation in Kim-1, Timp-1, TNF-α, and P53 by 554.48%, 407.96%, 567.13%, and 390.47%. Furthermore, Figure 3 revealed elevated expression of Caspase-3, cleaved Caspase-3, and Keap-1 in the ACR group by 746.82%, 100.03% and 417.4% compared to the control. Our results revealed a significant upregulation (*p < 0.001*) of the Bax gene by 81.79%, while downregulation of the NRF-2 gene by 80.15% due to exposure to ACR compared to the control. Meanwhile, the results of our experiment revealed that the combination therapy with ZNO-MONPs and ACR caused significant inhibition of Caspase-3, cleaved Caspase-3, P53, TNF-α, Kim-1, Bax, and Keap-1 than that observed in the ACR group and elevated the expression of the NRF-2 gene by 308.13% regarding the consecutive levels in the ACR group.

### 3.5 Expression of miRNA 21a-5P and miRNA 122-55P

**FIGURE 4:**
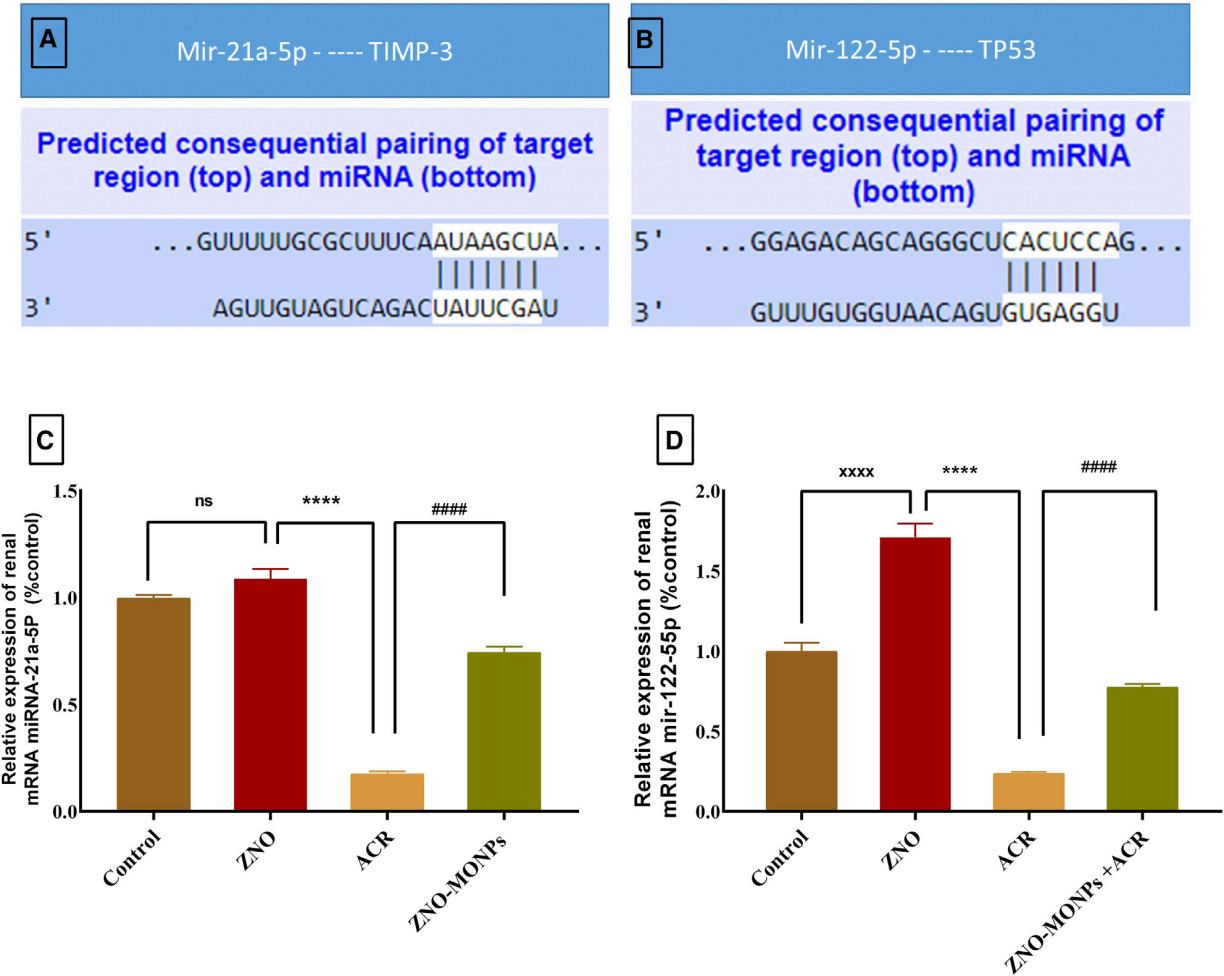
Represents the microRNA (miRNA) 21a-5P and 122-55P consequential pairing of target regions with Timp-1 **(A)** and TP53 **(B)** respectively. Also, the effect of Moringa olifera-mediated zinc oxide nanoparticles (ZNO-MONPs) and/or acrylamide (ACR) exposure on the expression of miRNA 21a-5P **(C)** and miRNA 122-55P **(D)** of all experimental groups including control of adult male *Sprague Dawley* rats for 60 days. Bars represent the mean ± SE. n = 10. ****p < 0.001 vs. control, #### *p* < 0.001 vs. ACR.

Figure 4 revealed the pairing sites between miRNA 21-a and Timp-3 (Figure 4A) and miRNA 122-55P with TP53 (Figure 4B). Exposure to ACR resulted in a substantial downregulation (*p < 0.001*) of miRNA 21a-5P and miRNA 122-55P expression by 82.46% and 76.25%, respectively. Conversely, the concurrent administration of green-synthesized Moringa nanoparticles with ACR1q in the combination group led to a notable reversal in upregulation, with an increase of 325.17% for miRNA 21a-5P and 227.85% for miRNA 122-55P. The presented data in the same Figure 4 demonstrates a significant difference in the expression pattern of these genes, showing upregulation of 325.17% and 227.85% for miRNA 21-a and miRNA 122-55P, respectively.

### 3.6 Histopathological findings

**FIGURE 5:**
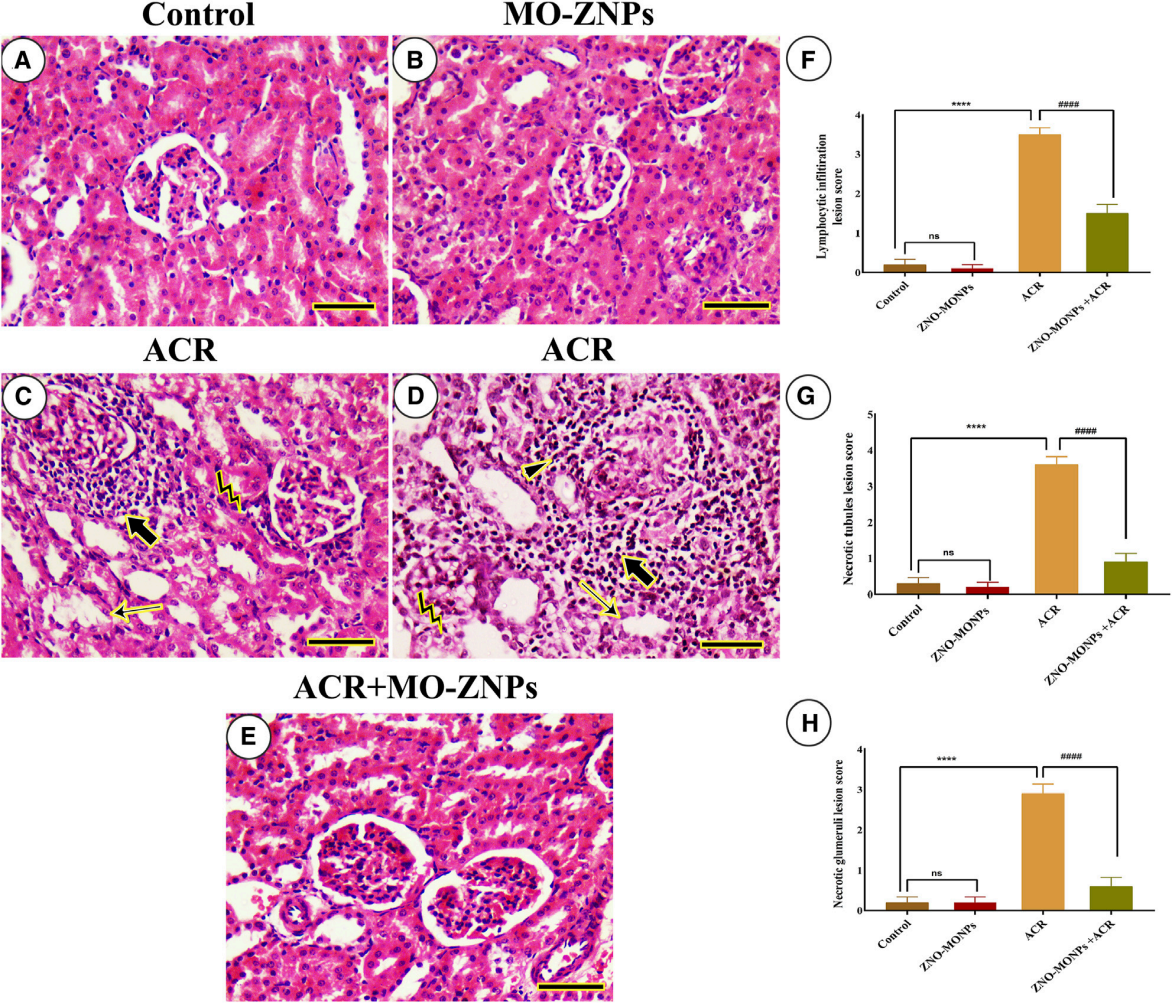
Histopathological examination of rat kidney. **(A)** Negative control. **(B)**, MO-ZNPs. **(C,D)** ACR group revealing excessive necrotic glomeruli (arrowhead) inside massive periglomerular inflammatory infiltrations (thick arrows), pyknotic nuclei (bent arrows), and necrotic renal tubules (thin arrows). **(E)**, ACR + MO-ZNPs. Scale bar = 50 mm. Moreover, the Lymphocytic infiltration lesion score **(F)**, Necrotic tubules lesion score **(G)**, and Necrotic glomeruli lesion score **(H)** in kidneys of all experimental groups including control of adult male *Sprague Dawley* rats for 60 days. Bars represent the mean ± SE. n = 10. ****p < 0.001 vs. control, #### *p* < 0.001 vs. ACR.

No histopathologic renal lesions were detected in the negative control and MO-ZNPs groups (Figures 5A,B). However, renal samples isolated from the ACR group showed necrotic glomeruli, necrotic renal tubules with several pycnotic nuclei, and massive lymphocytic infiltrations (Figures 5C,D). ACR + MO-ZNPs -treated rats improved the renal architecture in comparison to the ACR-only group (Figure 5E). The examined kidney fields were statistically analyzed for the detection of the degree of microscopical changes in the experimental groups and the ameliorative role of MO-ZNPs. The results revealed a significant (*p < 0.001*) elevation in the lymphocytic infiltration, necrotic tubules, and necrotic glomeruli lesion scores in the kidney tissue of the ACR group compared to the control. Meanwhile, the co-treatment with MO-ZNPs causes declined lesion scoring of lymphocytic infiltration by 57.14%, necrotic tubules by 75.0%, and necrotic glomeruli by 79.31% compared with the ACR-exposed group (Figures 5F–H).

### 3.7 Collagen distribution assessment of kidney

**FIGURE 6:**
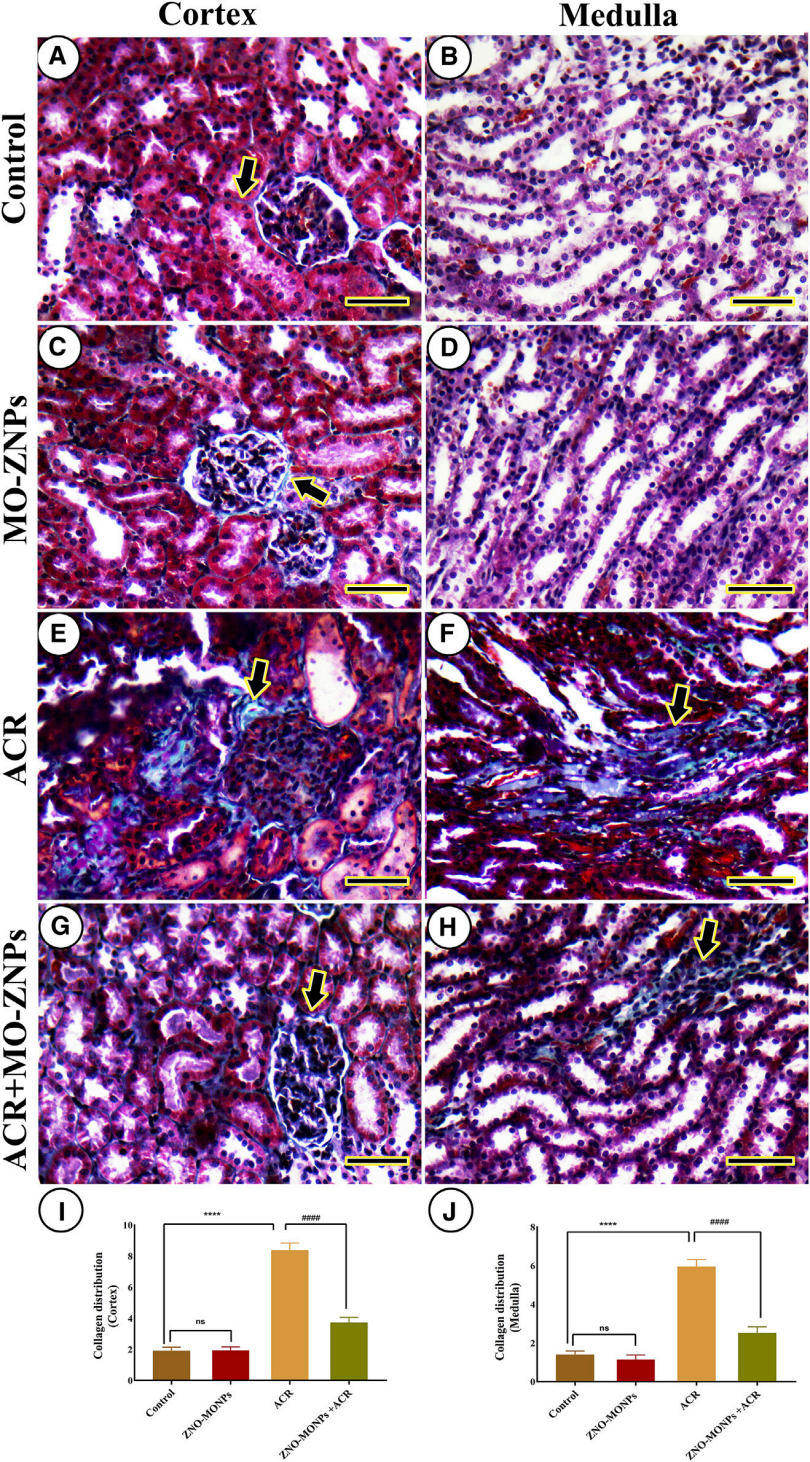
Histochemical staining of rat kidneys by Masson Trichrome technique. **(A, B)** Renal cortex and medulla of the negative control group, respectively. **(C, D)** the renal cortex and medulla of the MO-ZNPs group, respectively. **(E, F)** the renal cortex and medulla of the ACR group, respectively. **(G, H)** Renal cortex and medulla of ACR + MO-ZNPs group, respectively. Collagen histochemical reactions (thick arrows). Scale bar = 50 micrometers. **(I)** Collagen distribution score in the renal cortex, and **(J)** Collagen distribution score in the renal medulla. Bars represent the mean ± SE. n = 10. ** *p* < 0.001 vs. control, #### *p* < 0.001 vs. ACR.

The study of renal fibrosis through the investigation of collagen distribution in the cortex and medulla of different groups revealed a very mild collagen distribution in the negative control and MO-ZNP groups (Figure 6A–D). On the other side, the samples from ACR-treated rats showed the highest collagen distribution (Figure 6E,F). Rats treated with ACR and MO-ZNPs displayed a lower collagen distribution than the ACR group (Figure 6G,H). The nonparametric quantitative analysis for the area percentage of collagen distribution illustrated significantly elevated distribution in ACR-treated rats in comparison to control and MO-ZNPs rats. This distribution was markedly lowered in the ACR + MO-ZNPs group (Figure 6I,J).

### 3.8 Molecular docking

**FIGURE 7:**
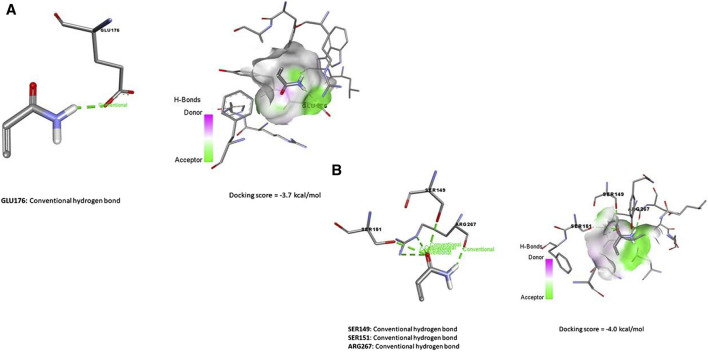
Molecular docking interaction of acrylamide (ACR) with **(A)** Bcl-2, and Glutathione synthase **(B)**.

**FIGURE 8:**
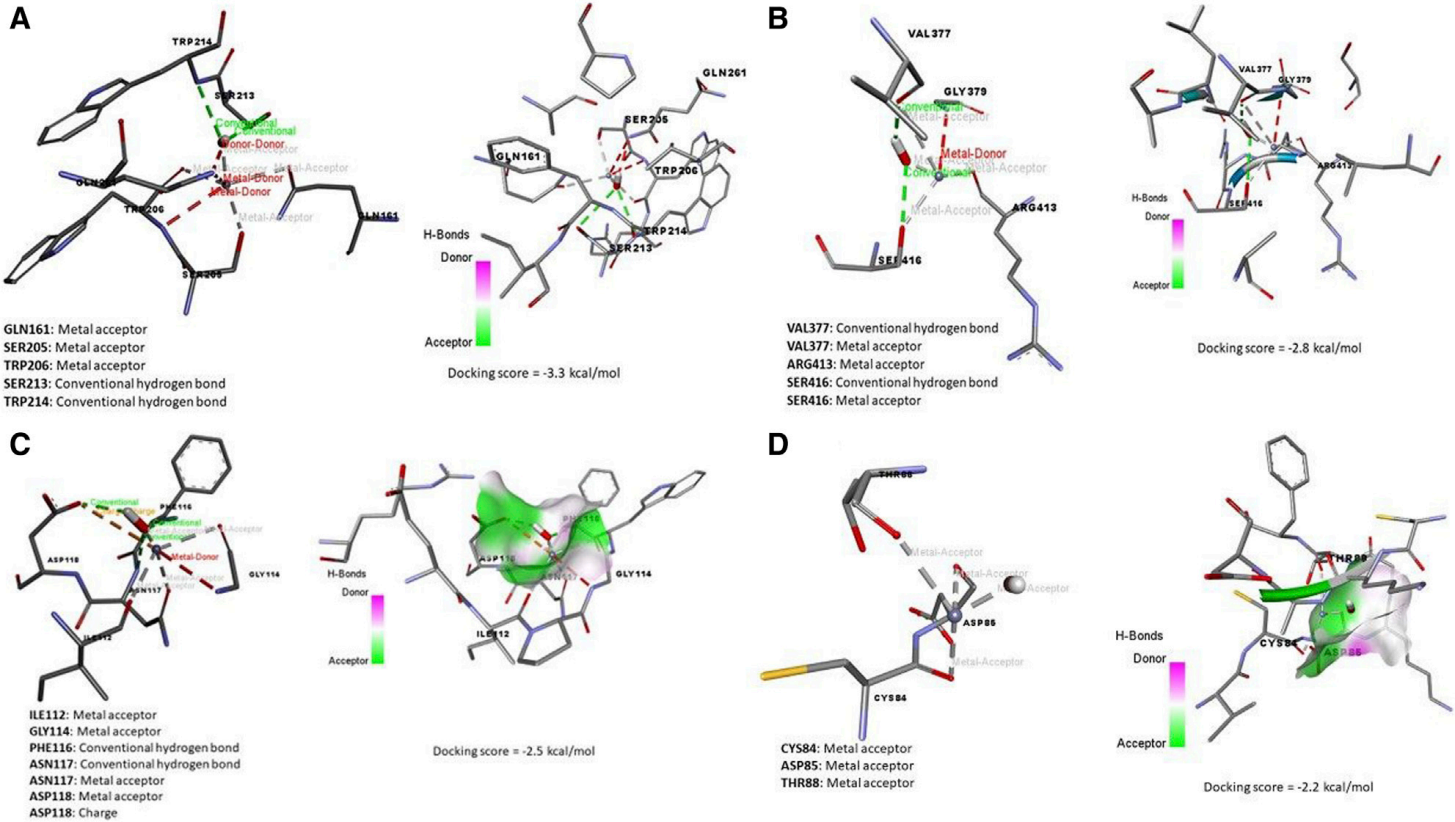
Molecular docking interaction of zinc oxide nanoparticles (ZNO) and **(A)** Caspase (3), **(B)** Kim-1, **(C)** Keap-1, and **(D)** TNFRSF1A TNF receptor superfamily member 1A (Tnfrsf1a).

Data represented in Figure 7A revealed the molecular docking interaction of ACR with GLU176 (Conventional hydrogen bond) residue in rats’ Bcl2 by energy of −3.7 kcal/mol. Also, ACR is bound to glutathione synthetase’s binding site by three conventional hydrogen bonds (SER149, SER151, and ARG267 residues) by −4.0 kcal/mol of energy (Figure 6B). ZNO interacted with caspase-3 by metal acceptor (GLN161, SER205, and TRP206 residues) and conventional hydrogen bonds (SER213 and TRP214 residues) by energy of −3.3 kcal/mol (Figure 8A). ZNO also, bound to the VAL377 (conventional hydrogen and metal acceptor), ARG413 (metal acceptor), and SER416 (conventional hydrogen and metal acceptor) residues in the Keap1 with −2.8 kcal/mol (Figure 8B).

**FIGURE 9:**
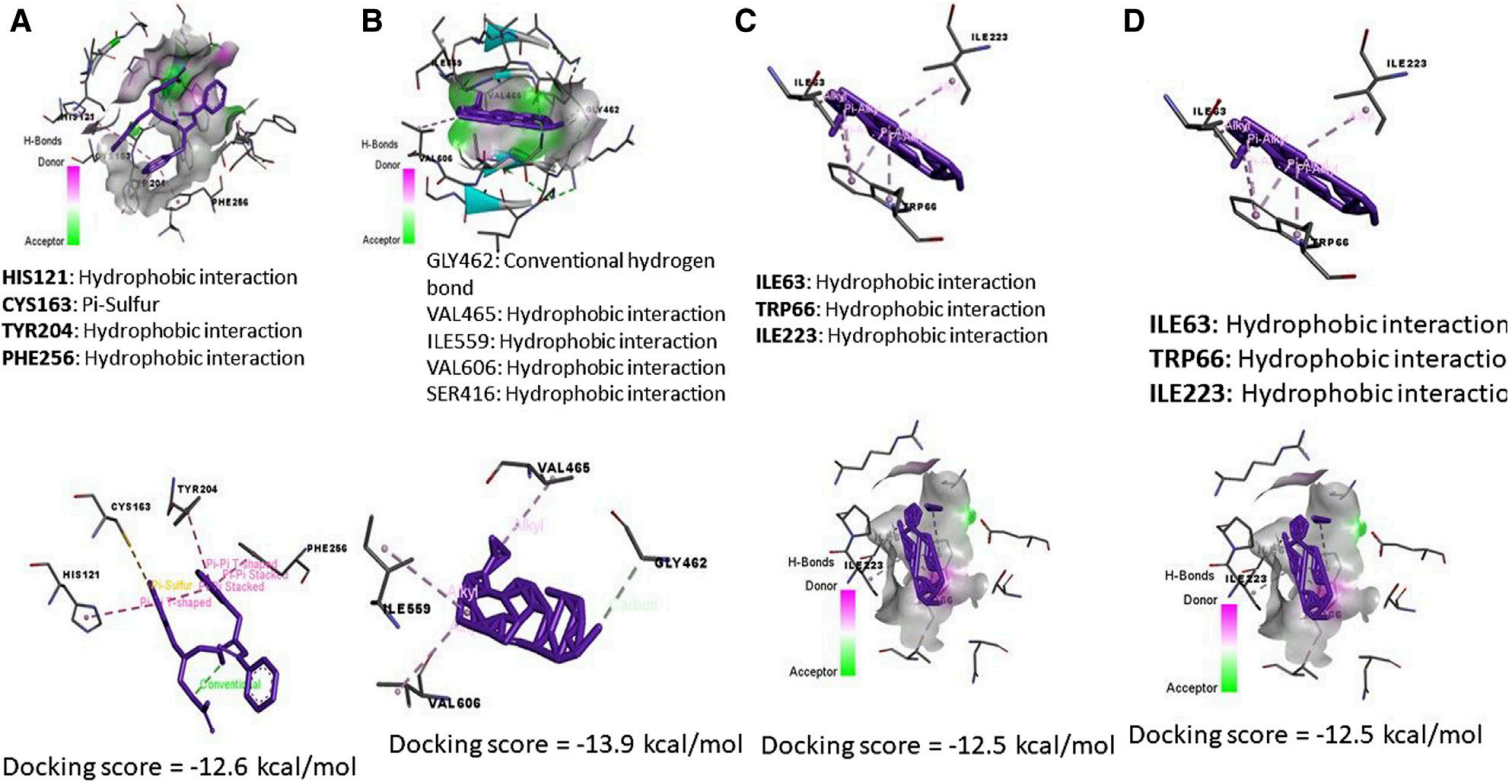
Molecular docking interaction of *Moringa olifera*-components (MONPs) and **(A)** Caspase (3), **(B)** Kim-1, **(C)** Keap-1, and **(D)** TNFRSF1A TNF receptor superfamily member 1A (Tnfrsf1a).

By the energy of −2.5 kcal/mol, ZNOinteracted with metal acceptor (ILE112, GLY114, ASN117, and ASP118) and conventional hydrogen bond (PHE116 and ASN117) residues in KIM-1 (Figure 8C). ZNO is bound by metal acceptor to CYS84, ASP85, and THR88 residues in the binding site of Tnfrsf1a with −2.2 kcal/mol energy (Figure 8D). Moreover, molecular interaction and docking scores of *MO* bioactive compounds against caspase-3, Keap1, KIM-1, and Tnfrsf1a are presented in Table 3 and Figure 8. Lupeol, the bioactive compound of *MO* exhibited a high affinity to inhibit caspase-3 (Figure 9A), Keap1 (Figure 9B), KIM-1 (Figure 9C), and Tnfrsf1a (Figure 9D) by the energy of −12.6, −13.9, −12.5, and −12.4 kcal/mol, respectively.

**TABLE 3:** Molecular interaction of M.oleifera’s bioactive compounds against Caspase-3, Keap1, KIM-1, and Tnfrsf1a.

Lotus IDs	Molecular docking scores (kcal/mol)			
	Caspase-3	Keap1	KIM-1	Tnfrsf1a
LTS0000536	−6.6	−7.5	−5.9	−5.3
LTS0000546	−6.4	−7.7	−5.4	−6.0
LTS0003946	−6.5	−8.3	−6.6	−6.1
LTS0004100	−6.5	−6.0	−5.6	−6.1
LTS0008238	−7.6	−6.8	−7.2	−7.2
LTS0008692	−6.6	−8.7	−6.3	−6.1
LTS0011830	−6.5	−8.7	−7.1	−6.2
LTS0012948	−6.5	−8.2	−6.8	−6.1
LTS0014061	−4.8	−7.0	−4.0	−4.3
LTS0025489	−6.7	−8.2	−6.9	−6.1
LTS0027395	−6.2	−5.7	−6.0	−5.7
LTS0027783	−6.5	−7.2	−6.7	−6.1
LTS0032845	−10.3	−10.4	−8.7	−8.4
LTS0036151	−6.7	−8.3	−6.4	−6.4
LTS0042292	−9.9	−10.7	−9.5	−8.4
LTS0045356	−6.5	−8.1	−6.9	−6.0
LTS0047684	−7.0	−7.0	−7.4	−6.3
LTS0052576	−5.2	−4.5	−4.9	−4.2
LTS0053796	−6.5	−8.3	−6.7	−6.2
LTS0056971	−6.7	−8.7	−7.1	−6.2
LTS0071224	−8.0	−8.7	−7.6	−6.5
LTS0073002	−6.5	−8.3	−7.0	−6.1
LTS0078356	−6.6	−8.7	−7.1	−6.4
LTS0091192	−7.4	−8.5	−6.8	−6.8
LTS0095888	−6.4	−8.0	−7.1	−6.1
LTS0102548	−6.2	−6.4	−5.7	−5.5
LTS0107789	−6.5	−8.2	−6.4	−6.1
LTS0108928	−6.4	−6.3	−6.5	−5.5
LTS0112234	−6.5	−8.7	−6.9	−6.2
LTS0112854	−6.7	−8.2	−7.1	−6.2
LTS0113064	−6.7	−8.2	−6.6	−6.3
LTS0114250	−5.8	−6.3	−4.8	−5.0
LTS0114831	−3.4	−4.3	−3.5	−3.4
LTS0114897	−6.8	−8.6	−7.5	−6.1
LTS0117299	−6.4	−8.3	−6.5	−5.9
LTS0119170	−5.0	−5.2	−5.8	−4.3
LTS0124811	−8.1	−10.0	−8.1	−7.3
LTS0128050	−4.9	−5.6	−5.0	−4.4
LTS0134583	−6.5	−8.3	−6.4	−5.9
LTS0135111	−4.2	−4.8	−4.0	−3.9
LTS0139858	−6.5	−7.7	−6.5	−6.1
LTS0142201	−8.1	−9.8	−8.0	−7.2
LTS0143145	−6.4	−7.2	−6.8	−6.0
LTS0143756	−6.5	−6.6	−6.1	−5.9
LTS0145111	−5.5	−6.7	−5.2	−5.0
LTS0145866	−6.0	−6.7	−5.2	−5.1
LTS0145991	−5.6	−6.3	−4.9	−4.5
LTS0147139	−6.7	−8.2	−7.2	−6.3
LTS0147581	−6.6	−8.8	−7.0	−6.3
LTS0156307	−6.6	−7.8	−7.0	−5.6
LTS0157023	−6.9	−8.6	−7.5	−6.0
LTS0159479	−6.5	−7.9	−6.0	−5.6
LTS0168132	−8.1	−8.0	−6.8	−7.1
LTS0174765	−4.1	−4.6	−4.0	−3.7
LTS0183541	−6.3	−8.0	−6.3	−5.8
LTS0184109	−6.4	−8.1	−6.0	−5.8
LTS0184698	−6.3	−7.7	−5.8	−5.7
LTS0187548	−6.5	−8.2	−6.8	−6.1
LTS0194325	−6.1	−6.0	−5.3	−5.6
LTS0195029	−5.5	−5.6	−4.3	−4.5
LTS0195312	−8.8	−10.7	−7.7	−7.9
LTS0196477	−8.0	−8.9	−8.5	−6.7
LTS0196523	−6.6	−6.7	−6.3	−6.0
LTS0198402	−6.4	−7.8	−4.8	−5.9
LTS0201798	−8.5	−8.3	−8.4	−6.8
LTS0202597	−5.2	−5.0	−4.9	−4.8
LTS0203599	−7.6	−9.7	−7.6	−7.2
LTS0204616	−8.0	−8.4	−8.6	−6.7
LTS0205122	−6.5	−8.3	−7.0	−6.2
LTS0207164	−9.0	−10	−7.8	−6.5
LTS0208375	−6.7	−8.3	−6.9	−6.2
LTS0209029	−6.1	−6.0	−6.9	−5.3
LTS0210036	−4.2	−4.8	−4.4	−3.8
LTS0210495	−6.6	−7.8	−6.8	−5.7
LTS0214194	−10	−12.9	−10.3	−8.9
LTS0214881	−6.5	−8.0	−6.4	−6.1
LTS0222857	−4.2	−5.5	−4.5	−4.0
LTS0224398	−4.1	−4.9	−4.5	−3.7
LTS0233992	−6.0	−6.2	−5.2	−4.9
LTS0239873	−6.8	−8.6	−7.4	−6.2
LTS0243016	−10.5	−10.8	−9.2	−8.3
LTS0243835	−4.5	−5.0	−4.5	−3.9
LTS0246008	−5.5	−5.4	−5.9	−4.9
LTS0247439	−6.4	−6.2	−5.3	−4.8
LTS0249588	−7.0	−7.5	−6.2	−6.1
LTS0249950	−6.3	−8.1	−6.8	−6.0
LTS0251929	−8.9	−8.7	−8.8	−8.0
LTS0254337	−8.8	−10.7	−8.9	−7.9
LTS0254798	−6.2	−8.2	−6.0	−5.8
LTS0256952	−12.6	−13.9	−12.5	−12.4
LTS0257845	−6.6	−8.7	−6.8	−6.2
LTS0259836	−3.9	−4.3	−3.7	−3.6
LTS0260576	−6.5	−8.3	−5.9	−6.2
LTS0267055	−6.9	−7.6	−6.7	−6.1
LTS0267945	−7.2	−7.5	−7.1	−5.3

Kelch Like ECH, Associated Protein 1(Keap-1), kidney injury molecule −1(Kim-1), and TNF, Receptor Superfamily Member 1A (Tnfrsf1a).

## 4 Discussion

Acrylamide is a common environmental contaminant that forms during the cooking process of certain foods, particularly those rich in carbohydrates (Krishnakumar and Visvanathan, 2014). Given its widespread occurrence in everyday diets, understanding its effects on vital organs, such as the kidneys, is crucial for public health. Several emerging pieces of evidence suggest that acrylamide may pose health risks, including carcinogenicity and neurotoxicity (Uthra et al., 2017). Investigating its impact on kidney tissue adds to our comprehension of potential adverse health effects, contributing to a comprehensive risk assessment. The kidneys play a pivotal role in maintaining physiological homeostasis, including filtration, excretion, and regulation of essential substances (Abdel-Rahman Mohamed et al., 2022b). Any disruption in renal function can have cascading effects on overall health (Abdel-Rahman Mohamed et al., 2022a). Understanding how acrylamide affects kidney tissue is vital for comprehending its broader implications on systemic health. Our results indicated that ACR caused elevation in the levels of creatinine, uric acid, and albumin in the serum of exposed rats compared to the control. The notable increase in creatinine and urea levels is indicative of compromised glomerular filtration and impaired renal excretory function (Couchoud et al., 1999). Elevated serum creatinine levels, in particular, suggest a reduced clearance of this waste product, reflecting diminished kidney filtration capacity (Mendelssohn et al., 1999). Similarly, the increased urea levels underscore impaired renal reabsorption and further support the evidence of renal dysfunction induced by acrylamide exposure. The elevated serum uric acid levels observed in our study are consistent with impaired renal handling of this end product of purine metabolism (Bin-Jumah et al., 2021; Rahman et al., 2022). Renal dysfunction often leads to decreased excretion of uric acid, resulting in its accumulation in the bloodstream (Bin-Jumah et al., 2021). The rise in serum albumin levels suggests alterations in the glomerular filtration barrier and increased permeability (Dane et al., 2013). The increased serum concentrations of creatinine, urea, uric acid, and albumin following acrylamide administration underscore the nephrotoxic effects of this compound. Our findings align with existing literature by Erdemli et al. (2019); (Yousef and El-Demerdash, 2006; Kandemir et al., 2020), emphasizing the consistency and clinical relevance of ACR-induced renal dysfunction.

In this study, ZNO-MONPs were synthesized using a green method with *M. oleifera* (MO) leaf extract. The research aimed to explore the therapeutic potential of an eco-friendly, rapid, and cost-effective approach to green synthesized ZNPs to mitigate renal toxicity induced by ACR in a rat model. Characterization of the biosynthesized ZNPs using DLS and TEM revealed spherical particles with an average size of approximately 164 nm (Dahran et al., 2023). The study demonstrated that ZNO-MONPs played a role in repairing renal tissue structure following ACR-induced renal injury, restoring it to a nearly normal state comparable to the control group. This protective effect was attributed to the antioxidative properties of ZNO-MONPs, mitigating the impact of ACR on rat kidneys. Additionally, the primary reason for the antioxidant potential of *M. oleifera* peptides (<1 kDa) has been attributed to their ferric-reducing antioxidant power and DPPH (2,2-Diphenyl-1-picrylhydrazyl) radical-scavenging effect, as reported by (Aderinola et al., 2019). The research aligns with the trend of utilizing ZNPs from natural sources, as seen in previous studies with *Pelargonium odoratissimum* leaf extract (Abdelbaky et al., 2022). Zinc is known to prevent iron from participating in reactive oxygen species (ROS) generation cycles or Fenton reactions, thereby safeguarding the lipid structure of membranes from oxidative damage. Additionally, ZNO-MONPs significantly restored depleted antioxidants and reduced the increase in malondialdehyde (MDA) levels in renal tissue following acrylamide (ACR) exposure. This protective effect against oxidative stress has been observed in other studies involving rotenone exposure (Akintunde J. et al., 2021) and cisplatin administration (Erfani Majd et al., 2021). Furthermore, previous research has suggested that green zinc nanoparticles (ZNPs) can enhance superoxide dismutase (SOD) activity and stimulate the production of metallothionein, a potent scavenger of hydroxyl radicals. Additionally, zinc has been shown to protect sulfhydryl groups from oxidation and maintain intracellular glutathione (GSH) levels. Thus, it can be concluded that both the ZNO nanoparticles and the Moringa oleifera extract contribute to the antioxidant potential of ZNO-MONPs. Metal oxides, including CuO-NPs and silver oxides, have also been explored for their biological protective and antioxidant properties in recent studies, suggesting a broader application of green-synthesized metal nanoparticles (Xu et al., 2022).

Our findings revealed a significant increase in ROS and MDA levels, indicating heightened oxidative stress. This elevation can be attributed to the pro-oxidative nature of acrylamide, which disrupts the delicate balance between ROS production and antioxidant defenses. Notably, the observed decrease in TAC underscores the compromised ability of the antioxidant system to neutralize oxidative insults. Consistent with our results, ACR was proven to induce oxidative stress in several organs, including the brain (Dahran et al., 2023), liver (Gedik et al., 2017; Mahfouz et al., 2023), and testicular tissue (Dahran et al., 2023). In contrast, oral administration of ZNO-MONPs effectively attenuated the level of ROS. It inhibited the accumulation of lipid peroxides, as indicated by reduced MDA levels and elevated TAC in the renal tissue of rats exposed simultaneously to ZNO-MONPs and ACR for 60 days. It was found that M.olifera extract has been shown to counteract the generation of ROS and safeguard the kidney, liver, brain, and blood of rats from oxidative stress exerted by lead exposure (Velaga et al., 2014) and protected rat’s testicular tissue damage of oxidative stress (Mostafa-Hedeab et al., 2023). This suggests that ZNO-MONPs, synthesized through a green approach, possess the ability to alleviate the oxidative stress induced by ROS by scavenging them. Consequently, this contributes to the enhancement of the microenvironment and the antioxidant potential within the kidneys of the combined treatment group.

The presence of bioactive compounds in *Moringa oleifera* extract, particularly polyphenols and flavonoids, contributes significantly to the enhanced therapeutic properties of zinc oxide nanoparticles (ZNO). Polyphenols and flavonoids are well-known antioxidants and possess anti-inflammatory properties (Mohamed et al., 2019). When combined with ZNO nanoparticles, these bioactive compounds act synergistically to augment the antioxidant and anti-inflammatory effects of the nanoparticles. Polyphenols and flavonoids scavenge free radicals, inhibit oxidative stress, and reduce inflammation, thereby protecting cells and tissues from damage (Abd-Elhakim et al., 2024). Additionally, these compounds have been reported to enhance the stability and biocompatibility of nanoparticles, facilitating their interaction with biological systems, and improving their therapeutic efficacy. The green synthesis of ZNO nanoparticles with Moringa oleifera extract not only binds the beneficial properties of these bioactive compounds but also offers a sustainable and eco-friendly approach to nanoparticle fabrication (Mahfouz et al., 2023). By utilizing natural resources and avoiding the use of harsh chemicals, green synthesis minimizes environmental impact and promotes safety for both manufacturing processes and subsequent biomedical applications.

Herein, the obtained results revealed upregulated expression of Keap-1, Timp-1, and Kim-1 while downregulating expression of NLRF-2 in the ACR-exposed group regarding the control. The pronounced elevation in Kim-1 and Timp-1 expression observed in our study provides compelling evidence of the adverse effects of ACR on kidney tissue. Kim-1, or kidney injury molecule-1, is a well-established marker associated with renal tubular injury (Bonventre, 2009). Increased Kim-1 expression typically signifies a response to cellular stress and damage in the renal tubules (Prozialeck et al., 2009). Similarly, Timp-1, or tissue inhibitor of metalloproteinase-1, is a recognized biomarker for tissue damage and inflammation, often associated with impaired kidney function (Zhang et al., 2006).

The concurrent upregulation of Kim-1 and Timp-1 indicates a robust response of the kidney tissue to ACR-induced injury. Collectively, these biomarkers serve as sensitive indicators, reflecting the extent and severity of renal damage (Galal et al., 2018; Mohamed et al., 2022). The observed upregulation of Keap-1 introduces an exciting dimension to our findings, suggesting a potential role in the dysregulation of NRF-2 (nuclear factor erythroid 2-related factor 2) signaling. Keap-1 is a key regulator of NRF-2, an important transcription factor that orchestrates the cellular antioxidant response. Under normal conditions, Keap-1 acts as a sensor for oxidative stress and facilitates the degradation of NRF-2 (Han et al., 2013; Derangula et al., 2022). However, its increased expression in response to ACR exposure suggests a potential disruption in this regulatory mechanism. The upregulation of Keap-1 may interfere with the normal degradation of NRF-2, leading to its reduced availability for antioxidant gene transcription (Han et al., 2013).

In light of the present results, the upregulation of inflammatory and apoptotic genes, including Caspase-3, P53, and TNF-α in the ACR-intoxicated group compared to the control group shed light on the intricate molecular mechanisms underlying acrylamide-induced nephrotoxicity. The overexpression of Caspase-3, a crucial executor of caspase in the apoptotic pathway, is closely correlated with the observed apoptosis induction. The activation of the caspase-activated cascade signifies a cascade of events leading to programmed cell death in response to acrylamide intoxication. Additionally, the upregulation of TNF-α, a key cytokine in inflammation control, underscores the role of inflammation in ACR-induced renal damage. Existing literature, such as the work by (Li et al., 2006), emphasizes TNF’s contribution to inflammatory processes and its potential negative impact on liver health. Our findings confirm that ACR may induce nephrotoxic events and renal damage through the activation of TNF-α and caspase cascades. Moreover, the pivotal role of P53 in the induction of apoptosis in renal tissue further strengthens the link between acrylamide exposure and apoptotic pathways, highlighting the multifaceted nature of ACR-induced nephrotoxicity.

In this study, the simultaneous administration of ZNO-MONPs with ACR in rats clearly demonstrated a reversal of renal tissue damage, showing notable anti-inflammatory and anti-apoptotic potential. Similar events have been observed in various studies. For instance, (Soliman et al., 2020), investigated the ameliorative effects of *MO* against oxidative stress and hepato-renal dysfunction induced by methotrexate. Abu-Zeid et al. (2021) recently found that eco-friendly selenium nanoparticles, utilizing *MO* and/or MO ethanolic leaf extract, mitigate melamine-induced nephrotoxicity by alleviating renal function impairments, oxidative stress, and apoptosis in rat kidneys. Furthermore, *MO* leaf extract has shown protective effects against interstitial kidney inflammation with fibrosis by down-regulating KIM-1 in TiO2NPs-induced male albino rats (Ahmed et al., 2020). The treatment with *MO* extract has been associated with increased secretion of cortisol, adrenaline, Treg cells, NK cells, and leptin, promoting anti-inflammatory cytokines and regulating the immune system (Abdel-Latif et al., 2018). Moreover, *MO* treatment reduced the expression of KIM-1, TIMP-1, and TNF-α in male *Sprague Dawley* rats induced by ML (Abd-Elhakim et al., 2021). TNF-α, a pro-inflammatory cytokine that stimulates IL-1 and IL-6, was downregulated by *MO* in Seabream (*Sparus aurata*), while activated TGF-β induced anti-inflammatory effects (Mansour et al., 2018) and was also confirmed in the current study. These findings collectively highlight the potential of ZNO-MONPs to counteract renal damage effectively through multifaceted anti-inflammatory and anti-apoptotic mechanisms.

The administration of ZNO-MONPs effectively normalized serum levels of creatinine, uric acid, and albumin. This normalization contributed to the mitigation of pathological changes in kidney tissue, unequivocally affirming the protective impact of ZNO-MONPs on the kidneys. Similar findings have been documented in prior studies, where decreased levels of serum creatinine, blood urea nitrogen, and proteinuria were observed. Additionally, typical pathological manifestations, including glomerular basement membrane thickening, mesangial proliferation, and interstitial atrophy, were alleviated in diabetic nephropathic rats (Zhang et al., 2006; Nadro et al., 2018; An et al., 2020; Su et al., 2020).

miR-122-5p has demonstrated its regulatory role in various contexts including diabetic nephropathy and other kidney diseases (Cheng and Wang, 2020). A recent study highlighted its interaction with the long non-coding RNA XIST in the regulation of inflammation and apoptosis during acute kidney injury (Cheng and Wang, 2020). In the context of similar conditions like chronic pancreatitis, miR122-5p was found to be under-expressed (Calatayud et al., 2017). In our study, we observed a significant decrease in the expression of both miR-122-5p and miR 21a-5p in rats exposed to acrylamide (ACR) compared to the control group. Notably, the co-administration of ACR with green-synthesized zinc oxide nanoparticles (ACR + ZNO-MONPs) led to substantial attenuation of inflammation and fibrosis when miR 21a-5p was overexpressed in the renal tissue in the combination group. Accumulating evidence revealed that examining the mechanisms of action of miRs, commonly investigated by researchers, revealed that miR-125a directly suppressed fibrosis in a mouse model of diabetic nephropathy and reversed the fibrotic effects of miR-125a (Hao et al., 2021). Published studies further identified miR-122-5p as being lowly expressed and targeting the transforming growth factor-β receptor-II, influencing the progression of skeletal muscle fibrosis (Sun et al., 2020). This supports the notion that ACR induces inflammatory and fibrotic pathways by inhibiting the expression of miR 21a-5p.

In a previous study, the authors identified TIMP3 as a critical negative regulator of renal TNFα activity. In the absence of TIMP3’s negative regulatory function, TNFα further triggers apoptosis and inflammation, as reported in various pathophysiological conditions (Ramesh and Reeves, 2002). Additionally, the renal vasculature exhibited prominent pathological changes in TIMP3-deficient mice in a TNFα-dependent manner. TIMP3 has previously been implicated in kidney injury by regulating tissue fibrosis (Kawamoto et al., 2006). In this study, we demonstrate that the activity of miRNA21a-5P targeting TIMP3 was inhibited in the kidneys of rats exposed to ACR, preceding the development of marked interstitial fibrosis. This suggests that ACR could mediate fibrosis and inflammation by inhibiting TIMP3 and downregulating miRNA21a-5P. Meanwhile, the expression of both miRNA21A-5P and miRNA 122-5P significantly increased upon giving ZNO-MONPs to animals concurrently with ACR after an 8-week experiment.

## 5 Conclusion

In conclusion, our study reveals significant alterations in renal tissue function, molecular expression and microarchitectures following subchronic exposure to ACR in rat models. The exposed rats exhibited a decline in antioxidant capacity coupled with elevated levels of MDA and ROS, indicating substantial oxidative stress. Our findings suggest that ACR induces renal tissue damage through the inhibition of expression of miR-21a-5p and miR-122-5p, it accelerates both fibrosis and inflammation in the renal tissue of rats. Notably, the green-synthesized *MO* using zinc oxide nanoparticles (ZNO-MONPs) emerge as a promising protective agent against ACR-induced renal alterations, impacting tissue microenvironments and architectures. The observed protective effects may be attributed to the activating potential of ZNO-MONPs antioxidant enzymes, anti-inflammatory properties, reduction in lipid peroxidation, and up-regulating the miR-21a-5p and miR-122-5p that inhibiting fibrotic and inflammatory pathways. Consequently, we advocate for individuals at high risk of ACR exposure to consider incorporating naturally synthesized compounds containing metals, such as ZNO-MONPs, to mitigate ACR toxicity. These findings underscore the potential of eco-friendly interventions in safeguarding renal health amid environmental exposures.

## 6 Study limitations

While our study provides valuable insights into the impact of ACR exposure on renal tissue and the potential protective role of ZNO-MONPs, it is essential to acknowledge certain limitations:

1. Experimental Model: The study utilized a rat model for long-term ACR exposure, and the findings may not perfectly replicate human responses. Variations in metabolic rates, physiology, and ACR metabolism should be considered when extrapolating results to human populations.
2. Mechanistic Understanding: While our study proposes potential mechanisms underlying ACR-induced renal damage and ZNO-MONP protection, the exact intricacies of these pathways may warrant further investigation.
3. ZNO-MONP Dosage: The protective effects of ZNO-MONPs were observed at a specific dosage (10 mg/kg b.wt). Further research exploring a range of dosages is necessary to establish optimal concentrations and assess potential dose-dependent effects.
4. Clinical trials: While our findings suggest potential benefits of ZNO-MONPs, transitioning from animal models to clinical applications requires careful consideration of safety, efficacy, and ethical considerations. Clinical trials are necessary to validate these effects in human populations.

Future research addressing these limitations will enhance the applicability and strength of the findings, ultimately advancing our understanding of eco-friendly interventions for mitigating renal damage associated with environmental exposure to ACR.

## Data Availability

The original contributions presented in the study are included in the article/Supplementary Material, further inquiries can be directed to the corresponding authors.
