# Supplementary material for: Acrylamide-targeting renal miR−21a−5p/Fibrotic and miR122-5p/ inflammatory signaling pathways and the role of a green approach for nano-zinc detected via in silico and in vivo approaches
Source: Front Pharmacol. 2024 Jul 17;15:1413844. doi: 10.3389/fphar.2024.1413844 (PMC11289894; doi:10.3389/fphar.2024.1413844)
Supplement: Supplementary file 1 [file DataSheet1.docx]

Acrylamide-targeting Renal miR−21a−5p/Fibrotic and miR122-5p/ Inflammatory Signaling Pathways and the Role of a Green Approach for nano-zinc detected via In Silico and In Vivo approaches

Leena S. Alqahtani^1^, Manal E. Alosaimi^2^*, Amany Abdel-Rahman Mohamed^3^, Yasmina M. Abd-Elhakim^3^*, Tarek Khamis^4,5^, Ahmed E. Noreldin^6^, Ali H. El-Far^7^, Badriyah S. Alotaibi^8^, Mohammed Ageeli Hakami^9^, Naief Dahran^10^, Nouf A. Babteen^11^


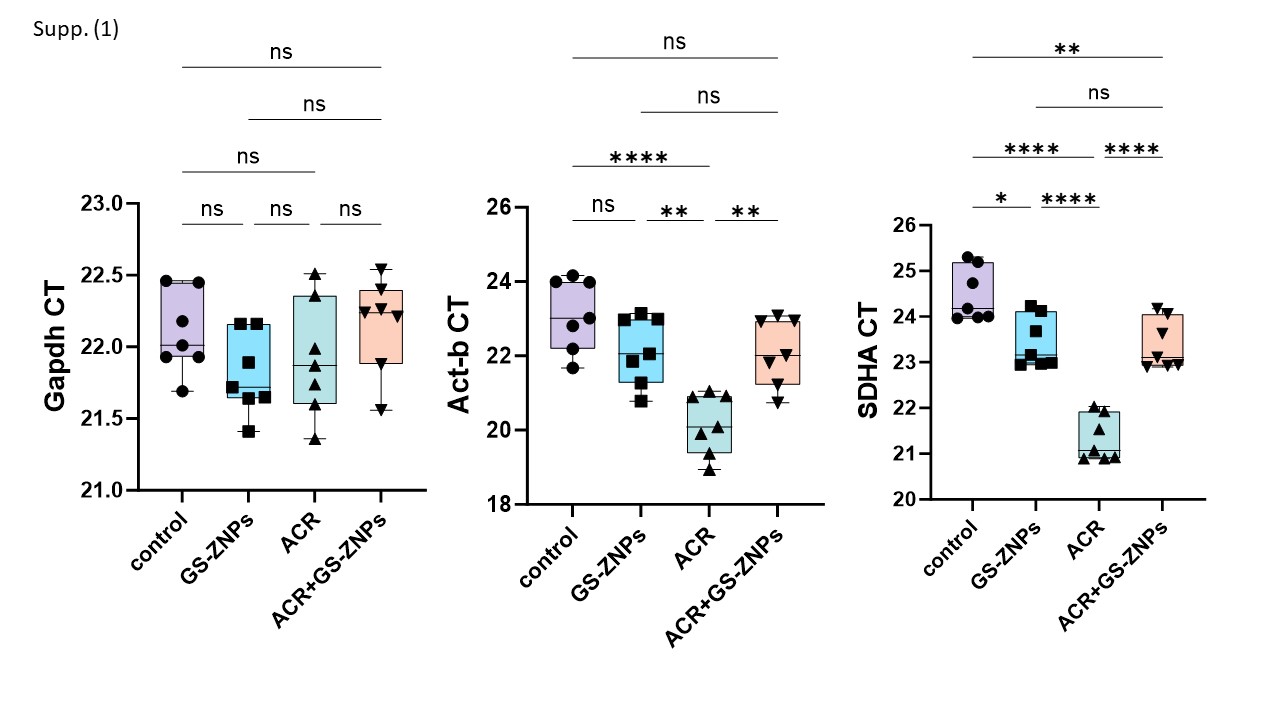


**Figure S1:** CT values of the house keeping genes GAPDH, actin-b, and SDHA. Bars represent the mean ± SD. n = 7.


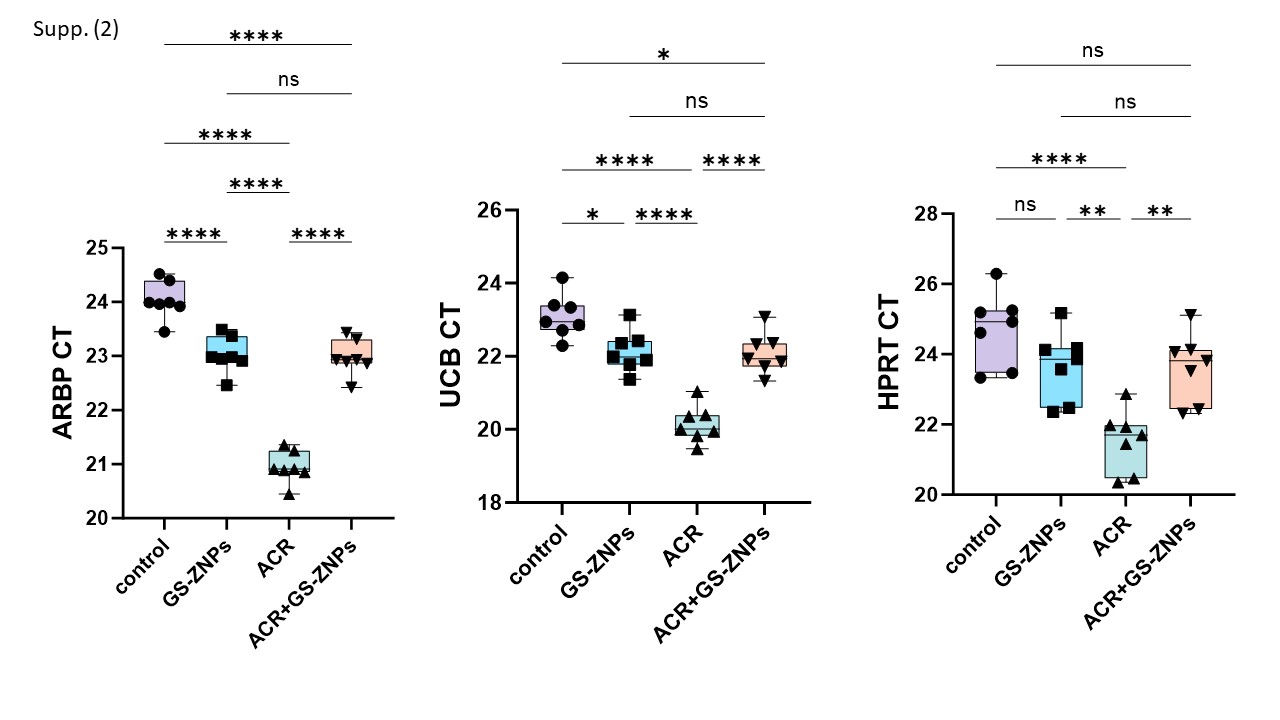


**Figure S2:** CT values of the house keeping genes ARBP, UBC, and HRPRT-1. Bars represent the mean ± SD. n = 7.
